# Bilateral Premammillary Artery Territory Infarctions After Clipping of a Ruptured Internal Carotid-Posterior Communicating Artery Aneurysm: A Case Report

**DOI:** 10.7759/cureus.107967

**Published:** 2026-04-29

**Authors:** Gosuke Endo, Yutaka Fuchinoue, Ryo Matsuzaki, Masaaki Nemoto, Nobuo Sugo

**Affiliations:** 1 Department of Neurosurgery, Toho University, Tokyo, JPN; 2 Department of Neurosurgery, Toho University, Chiba, JPN

**Keywords:** circuminfundibular plexus, clipping, covid-19, posterior communicating artery, premammillary artery

## Abstract

Preservation of the posterior communicating artery and its perforators is critical during clipping of posterior communicating artery aneurysms. We report the case of a woman in her 70s who developed bilateral premammillary artery territory infarctions after microsurgical clipping of a ruptured right internal carotid-posterior communicating artery aneurysm. On admission, her Glasgow Coma Scale score was 15, and her World Federation of Neurosurgical Societies grade was I. Intraoperatively, clip replacement and repeated readjustment were required. Final microscopic inspection and Doppler ultrasonography confirmed preservation of flow in the posterior communicating artery and its perforators. Postoperatively, she developed impaired consciousness and left hemiparesis, and serial imaging demonstrated bilateral low-density lesions in the premammillary artery territories. Muscle strength improved with rehabilitation, and she was transferred on postoperative day 20. The notable feature of this case was the bilateral distribution of infarction. We considered that, in addition to ipsilateral perforator compromise, reduced flow through the circuminfundibular plexus may have contributed to contralateral ischemia. This case highlights that bilateral perforator territory infarctions may occur despite apparent intraoperative preservation of perforator flow.

## Introduction

During clipping of posterior communicating artery (PCoA) aneurysms, preservation of both the PCoA and its perforators is essential for a favorable neurological outcome. The microanatomy of PCoA perforators has been described in detail, and the premammillary artery is regarded as one of the most constant and clinically important branches. It usually arises from the PCoA, although its origin, course, branching pattern, and anastomoses show substantial anatomical variation [1-5]. The premammillary artery supplies the posterior hypothalamus, mammillothalamic tract, anterior thalamus, inferomedial portion of the caudate head, genu, and part of the posterior limb of the internal capsule, and infarction in this territory may cause impaired consciousness, confusion, memory or behavioral changes, and motor deficits depending on the extent of internal capsule involvement [1,6]. Post-clipping premammillary artery infarction is an established but uncommon complication. In patients with unruptured PCoA aneurysms, larger aneurysm size and medial projection have been identified as independent risk factors for premammillary artery infarction [6]. In ruptured PCoA aneurysms, posterior projection has been associated with greater intraoperative difficulty, including rupture, use of fenestrated clips, dense adhesion to the PCoA or its perforators, and procedure-related complications [7].

Because bilateral thalamic infarction also raises the differential diagnosis of artery of Percheron infarction, this possibility should be considered when bilateral lesions are encountered [8]. We report a case of bilateral premammillary artery territory infarctions after clipping of a ruptured right internal carotid-posterior communicating artery aneurysm. The key feature of this case was the bilateral distribution of ischemia despite apparent intraoperative preservation of flow in the PCoA and its perforators.

## Case presentation

**Figure 1:**
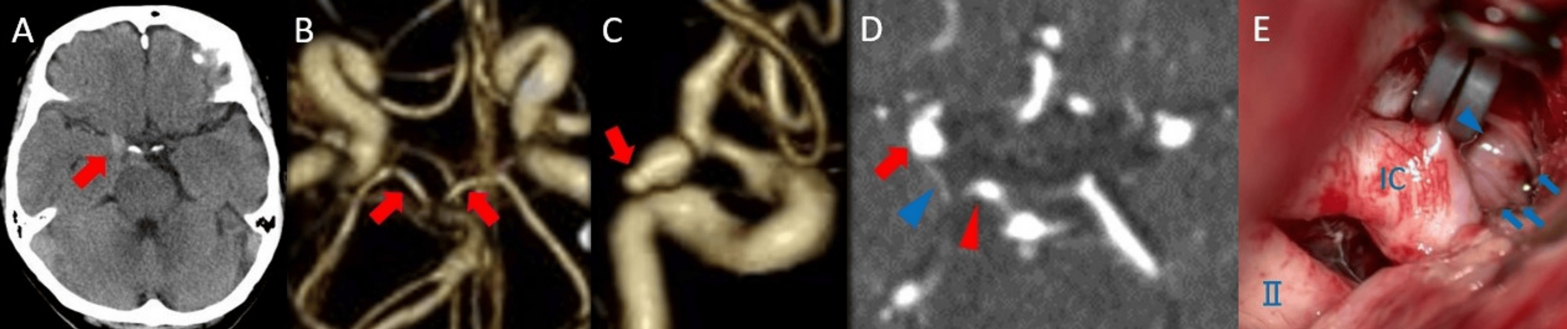
Preoperative imaging and intraoperative findings. (A) Initial non-contrast computed tomography (CT), axial view, demonstrating subarachnoid hemorrhage in the basal cistern (arrow). (B) Initial reconstructed computed tomography angiography (CTA), caudal view, showing well-developed bilateral P1 segments (arrows). (C) Initial reconstructed CTA, lateral view, demonstrating a bleb at the tip of the aneurysm (arrow). (D) Initial CTA, axial view, showing the posterior communicating artery (PCom) arising posteriorly from the internal carotid artery (red arrow), as indicated by the blue arrowhead. The PCom is smaller in caliber than the P1 segment, indicated by the red arrowhead. (E) Intraoperative microscopic view after clip application, confirming preservation of blood flow in the PCom perforators (arrows). The arrowhead indicates the PCom. IC = internal carotid artery; II = optic nerve

A woman in her 70s with a medical history of hypertension, asthma, and osteoporosis visited a previous hospital because of a fever and was diagnosed with coronavirus disease 2019 (COVID-19). The following day, she developed a sudden headache. Five days later, she revisited the previous hospital, where a subarachnoid hemorrhage was diagnosed, and she was transferred to our institution. On admission, her Glasgow Coma Scale (GCS) score was 15 (E4V5M6) [9], and she had right ptosis, diplopia, and right mydriasis without limb weakness. Her World Federation of Neurosurgical Societies grade was I [10]. Initial non-contrast computed tomography (CT) showed subarachnoid hemorrhage in the basal cistern (Figure 1A). Preoperative computed tomography angiography (CTA) demonstrated well-developed bilateral P1 segments (Figure 1B). The aneurysm measured approximately 7.5 mm in height and 4.4 mm in width, and a bleb was present at the aneurysm tip (Figure 1C). CTA also showed that the PCoA arose posteriorly from the internal carotid artery and was smaller in caliber than the ipsilateral P1 segment, indicating that the PCoA was present preoperatively and was not of fetal caliber (Figure 1D). The aneurysm appeared to arise from the internal carotid artery at the PCoA junction rather than from the PCoA itself.

Microsurgical clipping was performed under general anesthesia. The patient was placed in the supine position with the head elevated 15°, vertex down 10°, and rotated 30° to the contralateral side. After craniotomy, the dura was opened in an arcuate fashion, and the sylvian fissure was dissected to expose the aneurysm. Initially, a Sugita aneurysm clip No. 2 (straight, blade length 10.0 mm; MIZUHO Corporation, Tokyo, Japan) was used, but its reach was insufficient. It was then replaced with a Sugita aneurysm clip No. 13 (35° bayonet, blade length 10.0 mm; MIZUHO Corporation, Tokyo, Japan). Because intra-aneurysmal flow remained on Doppler ultrasonography, the clip was exchanged again for a Sugita aneurysm clip No. 14 (35° bayonet, blade length 12.0 mm; MIZUHO Corporation, Tokyo, Japan). During this process, the tip of the No. 14 clip caught a perforator, and several repositioning attempts were required. Final microscopic inspection confirmed that no perforator was trapped, and Doppler ultrasonography confirmed preserved flow in the PCoA and its perforators (Figure 1E). No intraoperative hypotensive episode was recorded.

**Figure 2:**
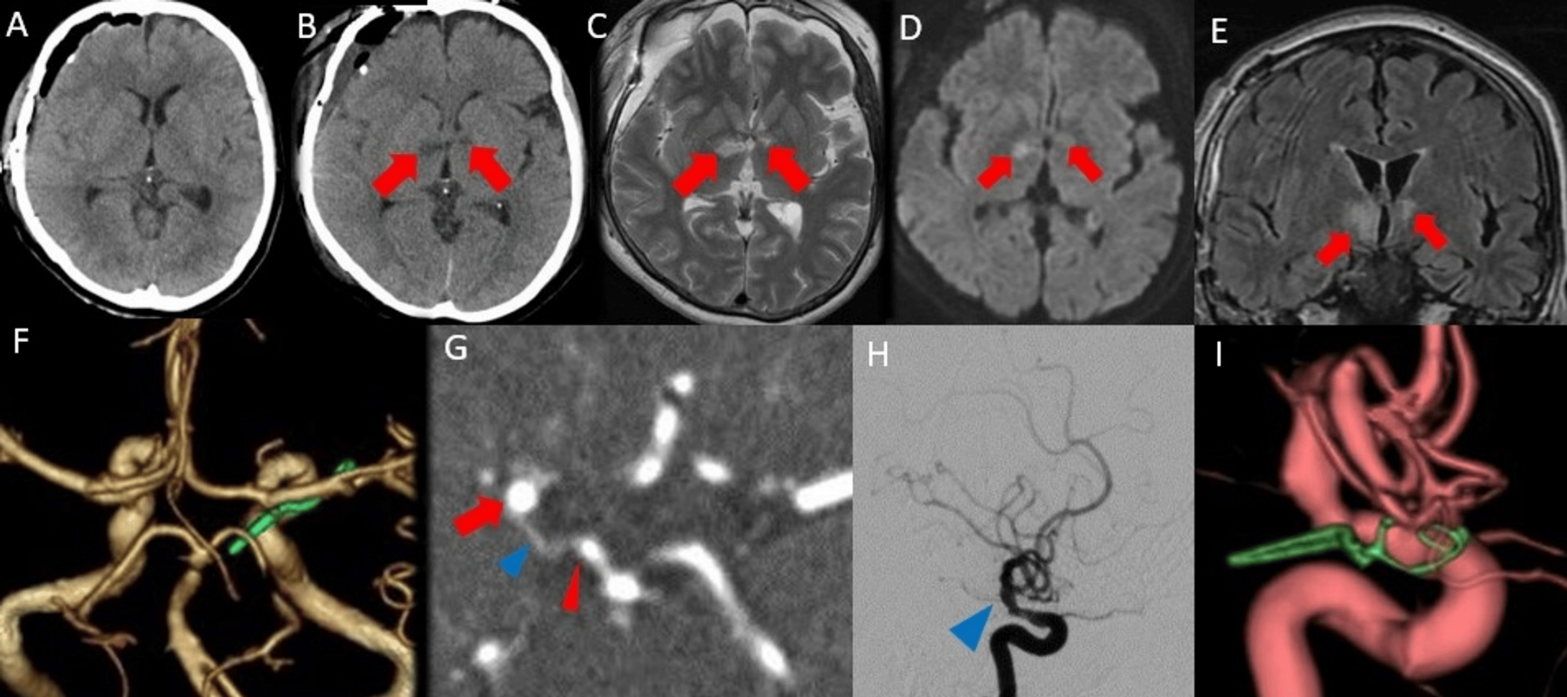
Postoperative imaging findings. (A) Non-contrast computed tomography (CT) obtained on postoperative day one, axial view, showing no apparent abnormality in the brain parenchyma. (B) Non-contrast CT obtained on postoperative day three, axial view, demonstrating low-density areas in the bilateral ventral thalami (arrows). (C) T2-weighted magnetic resonance imaging obtained on postoperative day 11, axial view, demonstrating infarcts in the same bilateral ventral thalamic regions. (D) Diffusion-weighted imaging obtained on postoperative day 11, demonstrating bilateral diffusion-restricted lesions corresponding to the abnormalities seen on the T2-weighted and fluid-attenuated inversion recovery images. (E) Fluid-attenuated inversion recovery magnetic resonance imaging obtained on postoperative day 11, coronal view, demonstrating infarcts in the same bilateral ventral thalamic regions. (F) Postoperative three-dimensional computed tomography angiography (CTA), superior view, showing that the tip of the clip does not extend to the contralateral side. (G) Postoperative three-dimensional CTA, axial view, showing the posterior communicating artery (blue arrowhead) arising posteriorly from the internal carotid artery (red arrow), as in the preoperative study. The P1 segment is also well visualized (red arrowhead). (H) Digital subtraction angiography obtained on postoperative day 17, lateral view, clearly demonstrating the posterior communicating artery (blue arrowhead). (I) Three-dimensional rotational angiography reconstructed image obtained from the same angiographic study, in which the posterior communicating artery is not clearly depicted on the three-dimensional reconstruction.

Because of a high COVID-19 viral load, extubation was delayed until postoperative day three. After extubation, her GCS score was E3V5M6 and later improved to E4V5M6 on postoperative day four [9]. She developed left hemiparesis, and manual muscle testing (MMT) showed grade 4/5 [11]. After rehabilitation, muscle strength improved gradually and reached MMT 5/5 before transfer on postoperative day 20. Non-contrast CT on postoperative day one showed no apparent abnormality in the brain parenchyma, whereas non-contrast CT on postoperative day three demonstrated low-density lesions in the bilateral ventral thalami (Figures 2A, 2B). T2-weighted magnetic resonance imaging (MRI) on postoperative day 11 demonstrated infarcts in the same bilateral ventral thalamic regions (Figure 2C). Diffusion-weighted imaging obtained on postoperative day 11 demonstrated bilateral diffusion-restricted lesions in the same regions (Figure 2D). Fluid-attenuated inversion recovery MRI on postoperative day 11, coronal view, also demonstrated infarcts in the same bilateral ventral thalamic regions (Figure 2E). Postoperative three-dimensional CTA, superior view, showed that the clip tip did not extend to the contralateral side (Figure 2F). Postoperative three-dimensional CTA, axial view, demonstrated that the PCoA still arose posteriorly from the internal carotid artery, as in the preoperative study, and that the P1 segment remained well visualized (Figure 2G). Digital subtraction angiography on postoperative day 17 clearly demonstrated the PCoA on the lateral projection (Figure 2H). In contrast, the PCoA was not clearly depicted on the three-dimensional rotational angiography reconstruction obtained from the same angiographic study (Figure 2I). These findings indicate that the PCoA was present both preoperatively and postoperatively and that poor depiction on the reconstructed three-dimensional image did not represent true loss of the vessel. Her modified Rankin Scale score was 4 at the time of transfer [12]. She was transferred to a rehabilitation hospital on postoperative day 20. At the three-month follow-up, she was asymptomatic and remained under outpatient follow-up. No definite right-sided motor deficit was documented. No clear postoperative neuropsychiatric symptoms such as apathy, abulia, anterograde amnesia, or executive dysfunction were recorded in the available chart.

## Discussion

The most notable feature of this case was the development of bilateral premammillary artery territory infarctions after clipping of a ruptured right internal carotid-posterior communicating artery aneurysm. Serial imaging showed no parenchymal abnormality on postoperative day one, followed by bilateral ventral thalamic low-density lesions on postoperative day three and corresponding infarcts on postoperative day 11 MRI. These findings indicate evolving bilateral ischemia in the early postoperative period.

The anatomical background is complex. The premammillary artery usually arises from the PCoA, but marked variability exists in its origin, number, course, branches, and anastomoses [1-5]. Its infarction can produce impaired consciousness, confusion, memory or behavioral disturbance, and weakness when the lesion extends to the internal capsule [1,6]. In our patient, the documented manifestations were impaired consciousness and left hemiparesis. No definite right-sided weakness or clearly documented thalamic neurobehavioral syndrome was identified. Previous clinical studies have shown that perforator-related ischemia after PCoA aneurysm clipping is influenced by aneurysm morphology and operative difficulty [6,7]. To facilitate clinical interpretation, the expected manifestations of premammillary artery territory infarction and the findings observed in the present case are summarized in Table 1 [1,6,8].

**Table 1:** Expected clinical manifestations of premammillary artery territory infarction and findings in the present case. GCS = Glasgow Coma Scale; MMT = manual muscle testing Expected findings were summarized based on previous anatomical and clinical reports [1,6,8].

Clinical domain	Expected findings in the premammillary artery territory infarction	Findings in the present case	Comment
Level of consciousness	Impaired consciousness may occur, particularly in acute bilateral involvement	Present	The patient showed postoperative impaired consciousness; GCS was E3V5M6 after extubation and improved to E4V5M6 on postoperative day four
Motor deficit	Contralateral weakness may occur when the lesion involves the genu or posterior limb of the internal capsule	Present	Left hemiparesis was observed and improved gradually with rehabilitation to MMT 5/5 before transfer
Bilateral motor symptoms	May be seen in bilateral lesions, depending on the extent and symmetry of internal capsule involvement	Not observed	No definite right-sided motor deficit was observed despite bilateral imaging abnormalities
Memory disturbance	May occur because of involvement of the mammillothalamic tract and the anterior thalamic region	Not observed	No anterograde amnesia was observed
Apathy/Abulia	May occur in thalamic-hypothalamic or anterior thalamic involvement	Not observed	No apathy or abulia was observed
Executive dysfunction/Behavioral change	May occur in thalamic infarction, especially when bilateral	Not observed	No executive dysfunction or other neuropsychiatric symptoms were observed
Laterality pattern correlation	Unilateral infarction may produce focal symptoms; bilateral infarction may cause broader mental status changes	Partially concordant	In this case, bilateral infarction was radiologically evident, but the observed clinical manifestations were mainly impaired consciousness and left hemiparesis

In the present case, the sequence of clip selection was surgically relevant. The change from a straight 10.0-mm clip to a 35° bayonet 10.0-mm clip and then to a 35° bayonet 12.0-mm clip effectively increased distal reach while preserving the working angle. Although this improved neck coverage, it also increased the possibility that the clip tip could extend toward perforators in a blind corner. The fact that the No. 14 clip tip transiently caught a perforator is consistent with this mechanical risk. Thus, when changing clip type or blade length in PCoA aneurysm surgery, the surgeon must reassess not only neck closure but also the precise direction and distal reach of the clip tip relative to adjacent perforators.

The most straightforward explanation for the ipsilateral lesion is transient or subtle perforator compromise during clip exchange and repeated repositioning. Even though final microscopic inspection and Doppler ultrasonography suggested preserved flow, short-lived compression, kinking, traction, or focal flow reduction near the clip tip could still have caused postoperative ischemia. However, this mechanism alone does not fully explain the bilateral distribution. Importantly, the PCoA was visible preoperatively on CTA, remained visible postoperatively on axial three-dimensional CTA and lateral digital subtraction angiography, and was not clearly depicted only on the three-dimensional rotational angiographic reconstruction from the same postoperative study. Therefore, the postoperative pattern is difficult to explain by simple PCoA loss or major-vessel occlusion alone.

We considered that reduced flow through the circuminfundibular plexus may have contributed to contralateral ischemia. The arterial supply to the pituitary stalk from above includes the superior hypophyseal, infundibular, and prechiasmal arteries [13]. Circuminfundibular anastomosis has been described as a potential anastomotic network around the pituitary stalk formed by these vessels bilaterally [14]. In this case, ipsilateral perforator compromise combined with hemodynamic disturbance in this peristalk anastomotic network may have contributed to ischemia in the contralateral premammillary artery territory. This remains a pathophysiological hypothesis rather than a directly proven mechanism. Artery of Percheron infarction is also an important differential diagnosis in bilateral thalamic infarction [8]. In the present case, however, the temporal relationship to unilateral PCoA surgery, the intraoperative episode of perforator impingement, and the preserved postoperative demonstration of the PCoA on CTA and lateral angiography favored a surgery-related perforator/hemodynamic mechanism over a primary artery of Percheron event, although an anatomical variant cannot be completely excluded.

Previous reports have also shown that apparent preservation of the parent artery or collateral filling does not necessarily guarantee the safety of perforator perfusion [15-17]. In addition, recent reports have described subarachnoid hemorrhage occurring during active COVID-19 infection and have emphasized that, although a temporal association may be present, a causal relationship between SARS-CoV-2 infection and subarachnoid hemorrhage remains unproven and controversial [18]. COVID-19 may induce systemic inflammation and coagulopathy, but these mechanisms are not uniformly demonstrable in individual cases [18]. Therefore, in the present case, the mention of COVID-19 should be interpreted as part of the perioperative clinical context rather than as evidence of a definitive COVID-19-related thrombotic or hemorrhagic mechanism. The practical lesson from this case is that bilateral perforator territory infarction may occur even when intraoperative findings suggest preserved PCoA and perforator flow. Thorough preoperative review of aneurysm morphology and the PCoA-perforator relationship, together with repeated multiangle reassessment during clip adjustment, is essential.

## Conclusions

We report a case of bilateral premammillary artery territory infarctions after clipping of a ruptured right internal carotid-posterior communicating artery aneurysm. The distinctive feature of this case was bilateral ischemia rather than infarction confined to the operative side. In addition to ipsilateral perforator compromise, reduced flow through the circuminfundibular plexus may have contributed to contralateral ischemia. Even when intraoperative inspection and Doppler ultrasonography suggest preserved perforator flow, postoperative perforator territory infarction may still occur.
